# Experienced Versus Perpetrated Intimate Partner Violence and Psychological Maladjustment: The Role of Adaptive and Avoidant Coping

**DOI:** 10.3390/ijerph22010036

**Published:** 2024-12-30

**Authors:** Julie L. Nagoshi, Craig Nagoshi, Farzana Akter, Vijayan K. Pillai

**Affiliations:** 1 School of Social Work, University of Texas at Arlington, Arlington, TX 76019, USA; pillai@uta.edu; 2 Department of Psychology, University of Texas at Arlington, Arlington, TX 76019, USA; atctn@asu.edu; 3 Department of Clinical Psychology, University of Dhaka, Dhaka 1000, Bangladesh; akter.farzana@du.ac.bd

**Keywords:** intimate partner violence, gender role beliefs, gender, coping, mental health

## Abstract

The present questionnaire study explores the relationship between intimate partner violence (IPV), coping strategies, and psychological maladjustment for both female and male college students, as well as considering the effects of perpetrated IPV. College students are at risk for experiencing and perpetrating IPV, and coping skills may act as important risk and protective factors. In total, 333 (247 women, 86 men) undergraduate college students completed an online survey for research participation credit. Perpetrated intimate partner verbal aggression and experienced and perpetrated relationship violence were significantly correlated with somatization and depression, while for women, perpetrated verbal aggression was significantly correlated with somatization, depression, anxiety, and eating disorder symptoms. Experienced verbal aggression was significantly correlated with depression for women, while perpetrated verbal aggression was significantly correlated with anxiety for men. Path analyses with bootstrapped mediation tests found that, for men, the relationships between experienced violence and somatization and depression were significantly mediated by avoidant coping, while for women, the relationships between perpetrated verbal aggression and somatization, depression, anxiety, and eating disorder symptoms were significantly mediated by avoidant coping. Findings suggest that, particularly for women, the use of avoidant coping behaviors may exacerbate cycles of victimization by and perpetration of IPV that, in turn, lead to greater psychological distress. The present findings suggest that interventions to reduce IPV should put greater emphasis on the teaching of adaptive coping skills in couple relationships to help reduce the impulse to perpetrate violence, on top of coping skills to deal with experienced violence.

## 1. Introduction

Intimate partner violence (IPV) has been shown to have multiple impacts on overall life course trajectory [1] and can be defined as emotional, physical and/or sexual violence between two partners [2]. Interpersonal violence, in fact, is the most commonly reported violence perpetrated against women [3], which includes intimate partner violence, dating violence, domestic abuse, child sexual abuse, child physical abuse, and sexual assault [4]. While IPV is typically experienced by women and represents the leading cause of death for women, it is also experienced in about one-third of men at a lower severity [2].

While it might be assumed that young adults, most of whom have only been in a few, if any, long-term romantic relationships, are at low risk for IPV, in fact, such violence has been found to be prevalent in college student samples [5,6]. For a sample of college women, Kaukinan et al. [5] found that, among those in casual dating relationships, 25% had been victims of dating violence, while 37% had perpetrated it, with these numbers being higher for those in long-term relationships. It is also notable that, for this population, reciprocal victimization by and the perpetration of IPV is common [6].

According to El-Serag and Thurston [7], people who have experienced intimate partner violence are at higher risk of multiple mental health problems, including anxiety disorders, eating disorders, post-traumatic stress disorder, and substance or alcohol abuse, as well as physical health problems, such as cardiovascular disease, chronic pain, sleep disturbances, gastrointestinal problems, and traumatic injuries. Kaukinan’s [6] review of studies of college student dating violence similarly found strong associations between dating violence and poorer mental health, as well as a protective effect of social support, an adaptive coping mechanism. The present study further explores the relationship of IPV and psychological maladjustment for both female and male college students by considering the mediating roles of adaptive and maladaptive coping behaviors, as well as considering the effects of perpetrated IPV.

### 1.1. Intimate Partner Violence and Coping Strategies

Several studies have shown the relationship between women’s experienced IPV and psychopathology [3,7,8]. Adaptive and maladaptive coping responses may play a key role in this relationship. Coping refers to the ability to make cognitive, emotional, and behavioral efforts to handle situations that overwhelm one’s resources [9,10]. In Lazarus and Folkman’s [11] theoretical framework on how stress arises and leads to psychological and physiological harm, coping, along with the cognitive appraisal of a situation as being stressful, mediates whether a stressful event will have negative impacts. Roth and Cohen propose that coping strategies can be divided into approach or avoidance coping [12]. Approach coping refers to the decision to manage the situation properly with a recognition of the potential danger or life stressor [12]. Studies have shown that this strategy is mostly used when the problem is considered less serious and the individual perceives that they have access to resources to deal with the problem [12]. Avoidance strategies refers to hiding, ignoring, or escaping the situation, which may result in further negative emotional and psychological consequences [12]. Avoidance coping is primarily used in situations that are perceived to be out of control or when individuals have limited access to coping resources. Reasons for not using adaptive coping include ignorance of adaptive coping methods, lack of social support, low self-efficacy, or low motivation [13]. Although it would be expected that adaptive approach coping would lead to lower psychopathology, while maladaptive avoidant coping would lead to higher psychopathology, results are sometimes mixed, suggesting other contextual factors are at work. For example, Mahmoud, Staten, Hall, and Lennie’s study of young adult college students found that, although female students used both adaptive and maladaptive coping more than male students, they had higher levels of anxiety [14]. While maladaptive coping significantly predicted depression, anxiety, and stress, adaptive coping was not a significant predictor of any of the three outcome variables. In contrast, among college students exposed to the trauma of a mass murder, the adaptive coping strategy of taking action was found to be associated with post-traumatic growth, i.e., a positive change after trauma [15].

It is notable that experienced IPV is associated with increased perceptions of stress in victims’ lives [16]. Survivors of intimate partner violence use various types of coping strategies to cope with their situations. Rizo’s qualitative study focused on coping strategies used by female IPV survivors to manage IPV and IPV-related stress, both while in the abusive relationship and after leaving [17]. Rizo found that these women used a variety of coping strategies and that some strategies were specific to the IPV [17]. It would be expected that the use of avoidant coping would exacerbate the effects of IPV, leading to psychological distress due to victims failing to recognize the abuse, address the causes, and implement appropriate solutions for relationship problems and distress.

Flanagan, Jaquier, Overstreet, Swan, and Sullivan found that avoidance coping did, in fact, mediate the relationships between psychological and sexual IPV victimization and post-traumatic stress disorder symptom severity, depression severity, and drug use problems among women survivors of intimate partner violence [18]. DeCou et al. found that trauma-related shame and trauma-coping self-efficacy significantly mediated the association between negative social reactions and PTSD symptoms for sexual assault victims [19], while Weiss et al. found that women who experience intimate partner violence were at heightened risk for drug use problems, which is linked to avoidance coping [20]. Weiss et al. suggest that women victimized by IPV may use drugs as an adaptive strategy to escape the negative affect, particularly when confronted with highly stressful situations [20]. Similarly, Flicker, Cerulli, Swogger, and Talbot found that women victimized by IPV who used disengagement, denial, and self-blame strategies of coping were more likely to experience symptoms of depression and PTSD [21]. These avoidant coping strategies may result from victims concluding that leaving the abuser may put the victim at risk for escalating violence, but such avoidant coping may prevent the use of more action-oriented coping strategies that could result in an end to the abuse or help the woman protect herself.

Other studies of the effects of adaptive vs. avoidant coping strategies on the psychopathology of IPV victims have yielded mixed results. Weiss et al.’s study of African American, Latina, and White IPV-victimized women, in general, found that avoidance coping was related to greater PTSD clusters, but effects of social support and problem solving coping varied across groups [22]. Similar mixed results for coping were found by Lilly and Graham-Bermann, where women low on emotion-focused coping had fewer PTSD symptoms than women who frequently used emotion-focused coping, but these individuals reported higher PTSD symptoms in the presence of frequent violence exposure [23].

### 1.2. Present Study

The present questionnaire/correlational study thus sought to build upon previous findings on the mediating role of adaptive and avoidant coping on the relationship between IPV victimization and mental health problems in a large, ethnically diverse college student sample that included both women and men. As noted, previous research has found a significant prevalence of IPV among college students [5,6]. The literature on the association between psychopathological disturbances and IPV-related experiences points to the role of coping mechanisms. Prior studies have found that maladaptive coping mechanisms in particular have a significant correlation with increasing psychopathological outcomes [24], particularly for victims of IPV [18,19,20,21]. Studies also suggest that adaptive coping can decrease psychopathological outcomes among victims of IPV [19].

The study was also explicitly designed to test and contrast these relationships in both men and women. While reviews of the literature indicate that IPV victimization and perpetration, including bidirectional IPV, are as prevalent for men as for women [25,26], the majority of studies of IPV’s effects on mental health have focused on women. Studies that include both genders, however, suggest that the effects of IPV vary by gender. For example, Caldwell, Swan, and Wood brown showed that the impact of intimate partner violence victimization is not equally distributed by gender [27]. For women, experienced IPV is associated with an increased risk of psychopathology. Ouellet-Morin and colleagues’ longitudinal study also found that women who were abused by romantic partners were at two-fold risk for new-onset depression, even after controlling for other risk factors [6]. Female IPV victims also disproportionately suffer from injuries, fear, and PTSD, as well as experiencing greater decreases in relationship satisfaction. In addition, Cater, Miller, Howell, and Graham-Bermann showed that childhood IPV was associated with higher levels of adult post-traumatic stress, anxiety, and non-suicidal self-harm in women compared to men, but no other main effects of gender on mental health were found [28].

In contrast, Ulloa and Hammett’s research found that the mental health outcome of IPV was gender-independent, with the highest severity of mental health problems being associated with victims of bidirectional IPV, regardless of gender [29]. A similar result was found by Próspero [30], who did not find significant gender differences in the relationship between severity of IPV victimization and mental health problems, such as anxiety, depression, hostility, and somatization.

This study also extends the existing literature by considering the relationships among experienced versus perpetrated IPV, coping, and mental health problems. The simultaneous focus on both the perpetration of and victimization by IPV reflects previous findings with college students that perpetration of IPV may be retaliation for victimization in a cycle of violence [31] and that IPV committed by women against men is often viewed as being more acceptable [32,33]. Ulloa and Hammett [29], in fact, found that such bidirectional IPV, regardless of gender, was associated with more severe mental health problems. We hypothesized that both experienced and perpetrated IPV will be associated with increased psychopathology and that these relationships will be significantly mediated by adaptive and avoidant coping. Exploratory analyses considered the gender moderation of these pathways.

## 2. Methods

### 2.1. Participants

The present study is a cross-sectional, questionnaire-based correlational design. All procedures were approved by the Institutional Review Board of the university. In total, 384 undergraduate psychology students (291 female, 92 male, 1 other) at a large southwestern state university completed an anonymous online survey for research participation credit. There were no specified inclusionary/exclusionary criteria. The majority of the participants identified as straight (86.7%), followed by bisexual (8.3%), gay or lesbian (2.1%), and other (1.8%). The mean age of the sample was 18.9 years (SD = 1.0) for women and 19.6 years (SD = 2.8) for men. The race/ethnicity of the sample was 24.8% Caucasian/White, 32.6% Hispanic/Latino, 26.9% Asian/Pacific Islander, 11.2% African American/Black, and 4.5% other. About half of the participants (53.0%) defined themselves as not currently dating, followed by steady or exclusive daters at 33.7%, occasionally dating at 10.9%, married 0.8%, with “other” 2.1%. Given the focus on gender differences in intimate partner relationships, results are reported only for participants who self-identified as straight (247 women, 86 men).

This sample is drawn from a previously published data set [34] that centered on positive and negative gender role beliefs and intimate partner violence, focusing on specific cultural beliefs of both feminine and masculine aspects of gender role beliefs such as marianismo and machismo and how these gender role beliefs interact with IPV.

### 2.2. Measures

In general, measures were chosen for being widely used in the research literature, including with college students, and for being easily administered in an online questionnaire. Reported Cronbach’s alphas are based on the present sample.

*Experienced relationship conflict* was measured by the 18-item Conflict and Tactics Scale [35], which asks about the extent to which someone experienced a behavior perpetrated on them by their romantic partner in the past year. This measure was chosen for its wide use in the IPV literature and ease of administration, but it has been criticized for being insufficiently comprehensive of different forms of IPV and for leaving too much room for interpretation of the items [36]. For those participants, not currently in a romantic relationship, the instructions asked them to report on their most recent past romantic relationship. Items 17 and 18 on knife or gun threats or use were not included in the present study, given the very low probability of anyone reporting such behaviors and given the potential of these items to upset participants. Items were responded to on a 7-point scale, with 0 = never, 1 = once, 2 = twice, 3 = 3–5 times, 4 = 6–10 times, 5 = 11–20 times, and 6 = more than 20 times. Items 4–8 (insulted or swore, sulked or refused to talk about an issue, stomped out of the room or house or yard, cried, did or said something to spite) were averaged to form an Experienced Verbal Aggression subscale. The percentage of participants reporting experiencing these behaviors at least once across these items ranged from 29 to 67 percent. The Cronbach’s alpha for this subscale was 0.83. Items 9–16 involved violent behaviors, including throwing things, pushing, hitting, and beating the person up. As the percentages of participants reporting these behaviors at least once across these items was very low, ranging from 3 to 13 percent, these items were recoded to just reflect having ever experienced the behaviors (1 vs. 0 for never), and then averaged to form an Experienced Violence subscale with a Cronbach’s alpha of 0.88.

*Perpetrated relationship conflict* was measured by modifying the 18-item Conflict and Tactics Scales [36] to ask about the extent to which the participant had perpetrated a behavior on their romantic partner in the past year. Again, for those participants not currently in a romantic relationship, the instructions asked them to report on their most recent past romantic relationship. Items 17 and 18 on knife or gun threats or use were again not included in the present study for the reasons given above. Items were responded to on a 7-point scale, with 0 = never, 1 = once, 2 = twice, 3 = 3–5 times, 4 = 6–10 times, 5 = 11–20 times, and 6 = more than 20 times. Items 4–8 were averaged to form a Perpetrated Verbal Aggression subscale. The percentage of participants reporting perpetrating these behaviors at least once across these items ranged from 32 to 69 percent. The Cronbach’s alpha for this subscale was 0.80. Items 9–16, involving violent behaviors, had very low percentages of participants reporting perpetrating these behaviors at least once across the items (2 to 6 percent), so these items were recoded to just reflect having ever perpetrated the behaviors (1 vs. 0 for never) and then averaged to form a Perpetrated Violence subscale with a Cronbach’s alpha of 0.89.

*Somatization* was measured by the Patient Health Questionnaire PHQ-15 Somatic Symptom Severity scale [37] and Spitzer, Kroenke, and Williams, α = 0.78 [38], which consists of 15 questions asking about how often one has been bothered by somatization symptoms, such as “stomach pain” and “dizziness”, in the past two weeks. Responses are coded 0 = “Not Bothered”, 1 = “Bothered A Little”, and 2 = “Bothered A Lot”. Scores are the sum of the responses across the 15 items.

*Depression* was measured by the Patient Health Questionnaire PHQ-9 Depression scale, α = 0.90) [39], which consists of 9 questions asking about the frequency of depressive symptoms one has experienced in the past two weeks, with items such as “Little interest or pleasure in doing things” and “Feeling down, depressed, or hopeless”. Responses are coded 0 = “Not at all”, 1 = “Several days”, 2 = “More than half the days”, and 3 = “Nearly every day”. Scores are the sum of the responses across the 9 items.

*Anxiety* was measured by the Patient Health Questionnaire GAD-7 Anxiety scale [40]; α = 0.82, which consists of 7 questions asking about the frequency of anxiety symptoms one has experienced in the past four weeks, with items such as “Feeling nervous, anxious, on edge, or worrying a lot about different things.” Responses are coded 0 = “Not at all”, 1 = “Several days”, 2 = “More than half the days”, and 3 = “Nearly every day”. Scores are the sum of the responses across the 7 items.

*Eating disorder symptoms* were measured by the Patient Health Questionnaire PHQ-ED Eating Disorder scale (α = 0.69) [38], which consists of 8 yes/no questions asking about the occurrence of behaviors indicative of a binge eating disorder. An example item is “Do you often feel that you can’t control what or how much you eat?” Scores are the number of yes responses across the 8 items.

*Coping* was measured using the Brief COPE [41], which consists of 14 two-item subscales that measure different coping responses. For the present study, participants were asked to report on their coping behaviors in the past year (indicate of a “coping style”) using a 4-point response scale ranging from 1 = “I haven’t been doing this at all” to 4 = “I’ve been doing this a lot.” Six of the subscales (active coping, emotional support, instrumental support, positive reframing, planning, acceptance) were combined (averaged) to form an adaptive coping scale (α = 0.85). Three of the subscales (denial, substance use, behavioral disengagement) were combined to form an avoidant coping scale (α = 0.62). While Carver states that the separate COPE subscales are not meant to be combined into necessarily adaptive vs. maladaptive avoidant coping, the above schema made sense in terms of the idea of the agency discussed above, and the alphas for the composite scales indicate that the separate COPE subscales did cluster within the composite scales [40].

## 3. Results

### 3.1. Data Analysis Plan

Gender mean differences on study variables were assessed by independent-samples *t*-tests and correlations among variables by Pearson correlation coefficients. Multi-group path analyses were conducted to test the significance of indirect pathways from experienced and perpetrated IPV to psychopathology through adaptive and avoidant coping.

### 3.2. Gender Mean Differences

Independent sample *t*-tests compared gender differences in experienced relationship verbal aggression and violence, perpetrated relationship verbal aggression and violence, adaptive and avoidant coping, somatization, depression, anxiety, and eating disorder symptoms (Table 1). Men scored significantly higher than women on experienced relationship verbal aggression and violence, while women scored significantly higher than men on somatization.

### 3.3. Correlations Between Experienced and Perpetrated IPV and Psycvhopathology

Table 2 presents the correlations between experienced relationship verbal aggression and violence, perpetrated relationship verbal aggression and violence, adaptive and avoidant coping, somatization, depression, anxiety, and eating disorder symptoms, separately by gender. It is notable that, for both men and women, experienced violence was almost perfectly correlated with perpetrated violence, i.e., severe IPV was almost always bidirectional. For men, perpetrated verbal aggression and experienced and perpetrated relationship violence were significantly correlated with somatization and depression, while for women, perpetrated verbal aggression was significantly correlated with somatization, depression, anxiety, and eating disorder symptoms. Experienced verbal aggression was significantly correlated with depression and anxiety for women, while perpetrated verbal aggression was significantly correlated with anxiety for men.

Some of these gender differences in correlations of IPV victimization or perpetration with psychological maladjustment were statistically significant, including experienced violence with somatization (Fisher’s *z* = 4.22, *p* < 0.001), perpetrated violence with somatization (Fisher’s *z* = 3.05, *p* < 0.01), experienced violence with depression (Fisher’s *z* = 2.73, *p* < 0.01), and perpetrated violence with depression (Fisher’s *z* = 2.70, *p* < 0.01).

### 3.4. Correlations Between Experienced and Perpetrated IPV and Coping

For both men and women, perpetrated verbal aggression and experienced and perpetrated relationship violence were significantly correlated with avoidant coping, with these correlations being notably higher for men than for women. For women, adaptive coping was significantly correlated with experienced and perpetrated verbal aggression.

### 3.5. Path Analyses of IPV, Coping, and Psycvhopathology

For both women and men, avoidant coping was also significantly correlated with nearly all of the psychological maladjustment measures, suggesting that avoidant coping might be significantly mediating the relationships between IPV victimization or perpetration and psychological maladjustment. To test this, a path analysis was conducted that modeled experienced relationship verbal aggression and violence and perpetrated relationship verbal aggression and violence as exogenous variables predicting adaptive and avoidant coping that, in turn, were tested as predictors of somatization, depression, anxiety, and eating disorder symptoms. Combined and separate analyses were also run for gender, with a difference chi-square calculated for the combined analyses to test for the equivalence of the estimated paths for the male versus female matrices. Mediation effects were tested using the bias-corrected bootstrapping of the confidence intervals option in Mplus version 5.21 [42] with maximum likelihood estimation using the covariance matrix. Missing data were handled using full information maximum likelihood.

**Figure 1:**
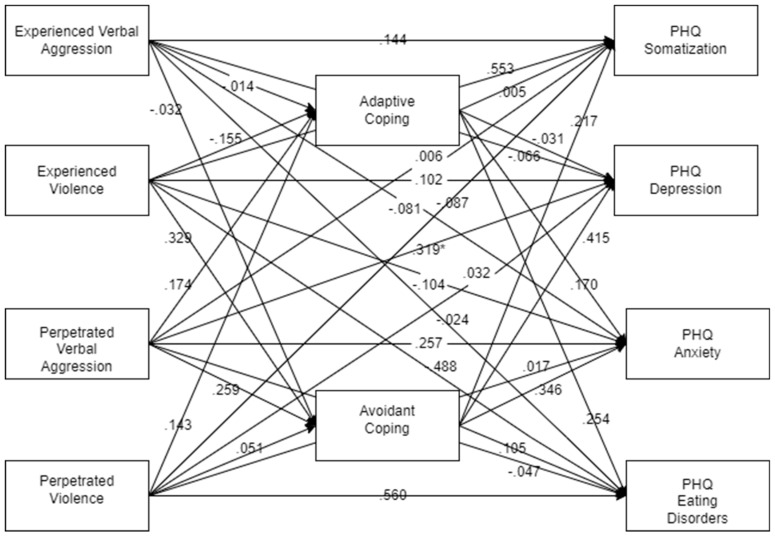
Path diagram for men showing standardized estimates. * *p* < 0.05.

**Figure 2:**
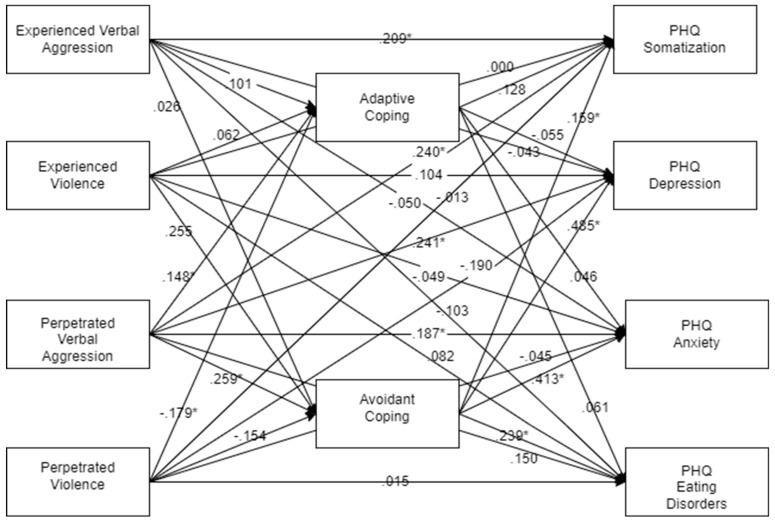
Path diagram for women showing standardized estimates. * *p* < 0.05.

Results of the path analyses are presented separately for the male and female (Figure 1 and Figure 2) estimates, as the difference chi-square for this model was highly significant (χ^2^ = 150.784, 45 df, *p* < 0.001, CFI = 0.795, RMSEA = 0.119). Bootstrapped mediation analyses indicated that, for men, the relationship between experienced violence and somatization and depression was significantly mediated by avoidant coping (95% confidence interval: 0.001, 3.552 for somatization; 0.028. 8.082 for depression). For women, the relationships between perpetrated verbal aggression and somatization, depression, anxiety, and eating disorder symptoms were significantly mediated by avoidant coping (95% CI: 0.010, 0.521 for somatization; 95% CI: 0.182, 1.537 for depression; 99% CI: 0.003, 0.932 for anxiety; 95% CI: 0.004, 0.054 for eating disorder symptoms). It is notable that none of the indirect paths between experienced IPV and mental health problems were statistically significant. None of the indirect paths involving adaptive coping were significant. It is notable here that significant mediated effects were found for the perpetration of but not victimization by IPV. When the direct and indirect paths from experienced vs. perpetrated IPV were set as equal to each other, the difference chi-square approached statistical significance (χ^2^ = 50.174, 36 df, *p* = 0.059, CFI = 0.975, RMSEA = 0.045), indicating that the causal relationships between IPV and psychopathology may differ for being victimized or acting as a victimizer.

## 4. Discussion

Consistent with previous studies [3,7,8] and our hypotheses, the current study of ethnically diverse college undergraduates found that experienced intimate partner violence was associated with an increased prevalence of mental health problems, including somatization, depression, anxiety, and eating disorder symptoms, with these relationships being found for both men and women. The relationships between experienced IPV and mental health problems in our study were found to be significantly mediated by avoidant coping only for men. This contrasts with studies like Flanagan et al. [18], which found that avoidant coping significantly mediated the relationship between experienced IPV and depression for a sample of women experiencing bidirectional IPV. While the lack of significant mediated effects of avoidant coping for experienced IPV for women could be attributed to possible low reliabilities of the measures, different measures across studies, and the younger college student sample, the correspondingly greater number of significant correlations of perpetrated IPV with mental health problems, and the several significant mediated effects of avoidant coping for these relationships for women, suggest that the role of coping in a relationship with IPV-related mental health problems needs to be understood through the lens of multiple factors leading to perpetrated IPV and recognizing gender differences in these causal pathways.

As noted above, the perpetration of IPV may be retaliation for previous victimization, resulting in a cycle of violence [6,31], and such retaliatory IPV may be more likely to be committed by women, as such violence against men is often viewed as being more acceptable [32,33].

The present study, in fact, found that reported victimization by violence, i.e., severe IPV, was highly correlated with perpetration of such violence for both men and women. Meanwhile, Ulloa and Hammett found that the highest severity of mental health problems was associated with victims of bidirectional IPV, and that this effect was consistent across gender [29]. Bidirectional IPV may lead to greater mental health problems because it perpetuates an escalating cycle of violence, but also because it may elicit feelings of guilt and inadequacy for intimate partners who commit violence against their partner. Sprunger, Eckhardt, and Parrott found evidence that the relationship between IPV victimization and perpetration was mediated by anger for women, but alcohol use for men, suggestive of gender differences in motivations for bidirectional IPV [43]. Meanwhile, an interview study of battered women conducted by Levine found that these women explicitly cited maladaptive coping skills as a factor in their engaging in bidirectional IPV [44].

Gender differences in the level of experienced and perpetrated IPV and the relationships among IPV, coping, and psychopathology may reflect long-established practices and beliefs with regard to gender and gender roles. Our previous published research from this sample showed that gender role beliefs associated with male domination and female submission in family relationships were positively correlated with experienced and perpetrated IPV, while gender role beliefs associated with family responsibility were negatively correlated with lPV [34]. The present findings suggest that interventions to reduce IPV should put greater emphasis on the teaching of adaptive coping skills in couple relationships to avoid the impulse to perpetrate violence, on top of coping skills to deal with experienced violence. Positive gender role beliefs emphasizing family responsibility and loyalty may be one avenue for fostering adaptive coping behaviors.

An often-cited frustration of clinicians who do identify IPV is that women tend to stay with their abusive partners [45]. While IPV victims cannot control their partner’s choice to use violence, they can change their response and they can act to increase their own safety [45]. For example, the targeting and control of anger responses between couples, such as anger displays and anger feelings, can also increase coping skills [46].

Prevention and intervention efforts can play a central role in increasing coping skills and coping strategies. One way to do this is to conduct a universal screening of IPV for anyone who is receiving healthcare and/or mental health services. Another key way to increase coping strategies is through dating violence prevention programs [47]. Dating violence prevention programs focus on increasing IPV knowledge and changing IPV attitudes, beliefs, and behaviors [48]. Violence prevention programs can work both at the individual level, relationship level, and at the community level, targetting both men and women. For example. Gibbs et al.’s [49] Stepping Stones and Creating Futures Intervention reduced men’s self-reported perpetration of IPV and strengthened women’s livelihoods, but not for women’s experiences of IPV, showing a need for increased targeted coping strategies and resources to be utilized with IPV populations. Research has shown multiple challenges to promoting behavioral change among men who perpetrate violence, such as social acceptance of IPV, hypermasculine attitudes, emotional problems, childhood exposure to violence, co-morbid mental health issues, and denial, minimization, and blame [50]. Graham et al.’s [51] review of seven programs targeted toward reducing IPV among male teens and young adults found evidence of effectiveness for only one, with programs typically focused on defining IPV and promoting non-violent norms for romantic relationship behaviors. While behavioral change and coping strategies can be fostered by strengthening group cohesion and therapeutic alliances, considering specific client mix and the applicability of course content, and building in opportunities for participants to witness personal growth [52], teaching adaptive coping behaviors may be another important component for successful intervention.

Lastly, IPV needs to be looked at from a bidirectional perspective including men as IPV perpetrators, as well as victims. Violence against men is largely under-reported, and research shows that male victims of domestic violence are many times reluctant to talk about what they experienced and to speak out about their ordeal due to fear of being ridiculed by others in society, such as their immediate family members, friends, and police officials [53,54]. Safe spaces should also be created for male victims of IPV, including providing resources that not only promote mental health coping strategies as victims, but also solution-focused strategies for reducing cycles of relationship violence perpetration and victimization.

### Limitations and Future Research

Limitations of this study include its cross-sectional design, which confounds inferences of causality, and the undergraduate psychology student sample, which limits the generalizability of the findings to more general populations. The survey itself depended on self-reporting, which may have created a social desirability bias. Men, in particular, may under-report committing abuse against their partners, due to awareness of societal condemnation of IPV [31]. As is typical in studies of IPV, the violence victimization and perpetration scales were highly skewed, such that, by definition, effects found with these scales are heavily based on outliers.

Another issue is the general nature of the coping measure used in the present study. The mixed findings on the mediating role of coping strategies in the IPV-psychopathology relationship suggest that context and the specific type of active vs. avoidant coping behaviors used are important determining factors [22,23]. Irving and Liu’s study of coping strategies used by woman survivors of domestic abuse found that the most prevalent strategies were in the placating and formal categories, with the latter involving such behaviors as encouraging the abuser to seek counselling or seeking refuge [55]. In addition, one can see some of the inherent problems of simplistically classifying these behaviors as active/adaptive vs. avoidant/maladaptive coping strategies. Future studies should focus on strategies specific to coping with both being victimized by and perpetrating IPV.

## Figures and Tables

**Table 1:** Means and standard deviations by gender.

	Gender		*t*
	Female	Male	
	*M* (*SD*)	*M* (*SD*)	
Experienced Verbal Aggression	0.70 (0.56)	0.90 (0.51)	2.81 **
Experienced Violence	0.17 (0.92)	0.64 (1.94)	2.87 **
Perpetrated Verbal Aggression	1.18 (1.13)	0.98 (1.09)	−1.38
Perpetrated Violence	0.18 (0.81)	0.44 (1.60)	1.85
Adaptive Coping	2.49 (0.70)	2.48 (0.78)	−0.11
Avoidant Coping	1.45 (0.54)	1.35 (0.47)	−1.39
PHQ Somatization	4.79 (5.37)	2.51 (4.16)	−3.39 ***
PHQ Depression	6.47 (7.23)	5.33 (7.19)	−1.20
PHQ Anxiety	3.71 (4.55)	2.86 (4.00)	−1.52
PHQ Eating Disorders	0.32 (0.47)	0.23 (0.42)	−1.55

** *p* < 0.01, *** *p* < 0.001.

**Table 2:** Correlations among variables ^a^.

	Exp. Verbal Agg.	Exp. Violence	Perp. Verbal Agg.	Perp. Violence	Adaptive Coping	Avoidant Coping	PHQ Som.	PHQ Dep.	PHQ Anx.	PHQ Eat. Dis.
Experienced Verbal Aggression		0.12	0.49 ***	0.14 *	0.16 *	0.16 *	−0.03	0.16 *	0.14 *	0.01
Experienced Violence	0.09		0.06	0.86 ***	−0.07	0.14 *	0.00	0.03	−0.02	0.12
Perpetrated Verbal Aggression	0.22	0.33 **		0.17 *	0.16 *	0.28 ***	0.21 **	0.38 ***	0.34 ***	0.16 *
Perpetrated Violence	0.06	0.97 ***	0.44 ***		−0.03	0.21 **	0.06	0.00	0.04	0.05
Adaptive Coping	0.06	0.03	0.16	0.07		0.11	0.17 *	0.06	0.12	0.10
Avoidant Coping	0.09	0.46 ***	0.30 *	0.39 ***	0.17		0.17 *	0.52 ***	0.45 ***	0.25 ***
PHQ Somatization	0.17	0.49 ***	0.28 *	0.42 ***	0.16	0.33 **		0.43 ***	0.47 ***	0.30 ***
PHQ Depression	0.04	0.36 **	0.54 ***	0.33 ***	0.09	0.45 ***	0.46 ***		0.68 ***	0.32 ***
PHQ Anxiety	−0.03	0.17	0.37 ***	0.07	0.27 *	0.37 **	0.30 *	0.72 ***		0.29 ***
PHQ Eating Disorders	−0.04	0.05	0.06	0.15	0.24 *	0.18	0.00	0.19	0.09	

Note. ^a^ Women above the diagonal and men below the diagonal. * *p* < 0.05, ** *p* < 0.01, *** *p* < 0.001.

## Data Availability

Collected data are in a data repository to insure confidentiality and can be requested through the corresponding author.
